# Antibiotic Stewardship for Canine and Feline Acute Urinary Tract Infection: An Observational Study in a Small Animal Hospital in Northwest Italy

**DOI:** 10.3390/antibiotics10050562

**Published:** 2021-05-11

**Authors:** Cristina Vercelli, Massimiliano Della Ricca, Mariachiara Re, Graziana Gambino, Giovanni Re

**Affiliations:** 1 Department of Veterinary Science, University of Turin, 10095 Grugliasco, TO, Italy; graziana.gambino@unito.it (G.G.); giovanni.re@unito.it (G.R.); 2 Centro Veterinario Torinese (CVT) Hospital, 10098 Rivoli, TO, Italy; mdellaricca@icloud.com (M.D.R.); mariachiararex@gmail.com (M.R.)

**Keywords:** antibiotic stewardship, urinary tract infection, dog, cat, local sensitivity

## Abstract

Antimicrobial stewardship programs (ASPs) have been suggested to reduce antimicrobial resistance phenomena in veterinary medicine, as antibiotics are commonly used without microbiological confirmation. The aim of the present study is to design a specific working flow for a tailored antimicrobial treatment in the case of canine and feline urinary tract infections (UTIs). Urine samples were collected by cystocentesis from 16 dogs and 12 cats presenting acute signs of UTI. The therapy was decided according to the minimal inhibitory concentration, and it was possible to monitor 14 dogs and 11 cats. Rescue therapy (amoxicillin and clavulanic acid) was included in emergency cases. *Escherichia coli*, *Proteus mirabilis*, and *Streptococcus canis* were isolated in dogs, and *Escherichia coli*, *Staphylococcus pseudintermedius*, and *Staphylococcus aureus* were isolated in cats. No multidrug-resistant strains were detected, but all *Staphylococci* were methicillin resistant. Only one cat received rescue therapy, and only one dog was recruited. Dogs were treated with tetracycline (1/14), fluoroquinolones (6/14), beta-lactams (6/14), and gentamicin (1/14), while cats received fluoroquinolones (3/11), nitrofurans (1/11), clindamycin (1/11), and beta-lactams (6/11). The success rate was very high. Our findings are interesting because this is the first ASP in Italy, and it may be used as a model to develop ASPs for other pathologies.

## 1. Introduction

The antimicrobial resistance (AMR) phenomenon is well known, and several control strategies are currently used worldwide, both for human and veterinary medicine [1]. Among all of these procedures, the antibiotic stewardship program (ASP) in small animal clinical practices is becoming more and more important [2,3]. This approach is focused on reducing inappropriate antimicrobial prescription, which is usually caused at the beginning of empiric treatment without an appropriate diagnostic procedure. This does not allow for the identification of the etiology of a pathology [3].

Urinary tract infections (UTIs) are among the most commonly diagnosed diseases in companion animals, which prematurely receive an empirical antibiotic treatment, frequently without confirmation by microbiological exam [4].

The extreme variability in antimicrobial resistance diffusion all over the world, especially considering multidrug-resistant (MDR) bacteria, is well described and underlines the importance of developing specific local antibiotic surveillance programs and prescribing guidelines [5,6].

Targeted therapy is a good tool to limit the spread of resistance in clinics and hospitals. It was previously reported that resistant bacteria can be transmitted from patient to patient through contact with medical and nursing staff to healthy animal carriers, and they can be maintained on surfaces [2]. These aspects are important from a public health point of view and could lead to the longer hospitalization of the patient, increased demand for diagnostic tests, and, of course, higher healthcare costs for owners [7]. As the release of new antimicrobial molecules in the next few years is possible, it is important firstly to consider that it is unlikely and, even if this does happen, people will be prioritized with the available drugs [8]. Moreover, European legislation will be increasingly stringent with respect to the use of antimicrobials in veterinary medicine [9]. Therefore, the use of these drugs will respect the well-established concept of rational and prudent use, and it will be necessary to justify their use through laboratory tests with the final aim to increase a tailored approach to the patients and their pathology. Several authors extensively explained the reasons for their concerns regarding the spread of antibiotic resistance. Therefore, many articles were written using a retrospective approach: A plethora of variables were taken into consideration to evaluate how this phenomenon can be limited [2,10]. Microbiological culture and antimicrobial susceptibility tests are considered the gold standard methods to achieve a correct diagnosis and to reach an individualized treatment based on a decision-making process [11,12,13].

It is known that the higher resistance frequency found in cases of UTIs in Southern European countries compared to Northern European countries is due to less severe regulations and poor surveillance programs for antibiotic prescription in companion animals [10]. According to all aforementioned factors, an improvement in surveillance and more conscious antibiotic prescription are required to reduce the AMR phenomenon in Southern European countries, such as Italy. Moreover, it was previously well established that clinical data have to be collected and must be correlated to clinical situations, as retrospective reports lack clinical correlation [6,10]

Veterinary medicine in which ASPs are still largely underdeveloped is a complex issue, and considering the important statements obtained by previous papers, the aim of the present study was to propose a specifically designed decision-to-make protocol to treat urinary tract infections in dogs and cats, with the final goal to prescribe antibiotics only in cases of bacterial isolation and with the specific indication of a susceptibility test, avoiding empirical treatment. Moreover, patients were monitored according to a control visit plan to elucidate if the treatment permitted them to find a clinical and microbiological cure and, if the therapeutic strategy failed, to establish if a reinfection or a recruitment occurred.

## 2. Results

### 2.1. Patients

In the present study, 16 dogs and 12 cats were enrolled. All of the patients presented specific signs and symptoms of a UTI. Among dogs, only one presented recruitment, while two dogs were euthanized within one month after the first visit due to the critical conditions caused by concomitant diseases (both were terminal cancer patients). None of them received empirical antibiotic treatment, and it was possible to schedule a complete follow-up according to the control visit plan. Among cats, none died, but it was not possible to follow up with one patient, due to of the owner’s decision to change veterinarians. Only one patient, in accordance with the planned rescue therapy, received an empirical treatment with amoxicillin and clavulanic acid, due to the serious clinical condition and the concomitance of other diseases: No bacteria were isolated, and the therapy was interrupted five days from the beginning of treatment. According to previous considerations, 14 dogs and 11 cats were considered for follow-up visits.

### 2.2. Susceptibility Tests

**Figure 1:**
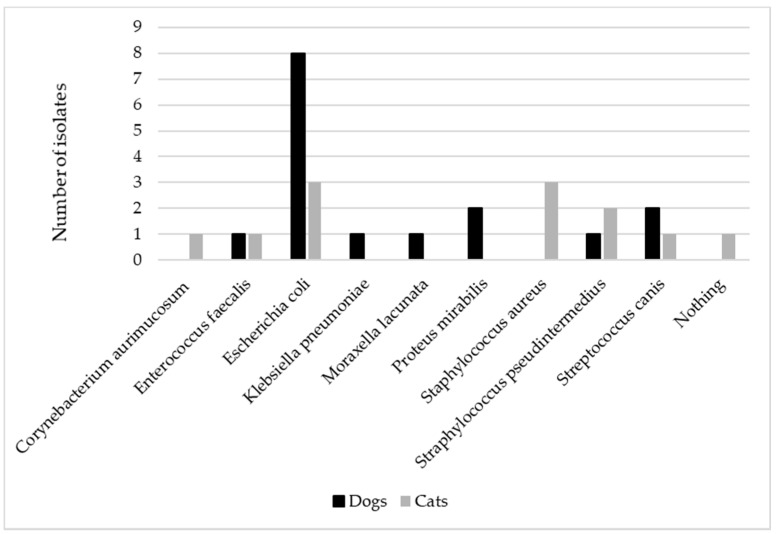
The figure represents the distribution of the different bacteria isolated in canine (*n* = 14) and feline (*n* = 11) urine samples collected by cystocentesis in patients presenting acute signs of urinary tract infection.

All samples were sent immediately to the laboratory and deemed adequate for carrying out the culture. No mixed infections were detected. *Escherichia coli* (*E. coli*) was isolated in most of the canine samples (8/14), followed by *Proteus mirabilis* (2/4) and *Streptococcus canis* (2/14), while *E. coli* (3/11), *Staphylococcus pseudintermedius* (3/11), and *Staphylococcus aureus* (2/11) were mainly identified among feline samples (Figure 1).

**Figure 2:**
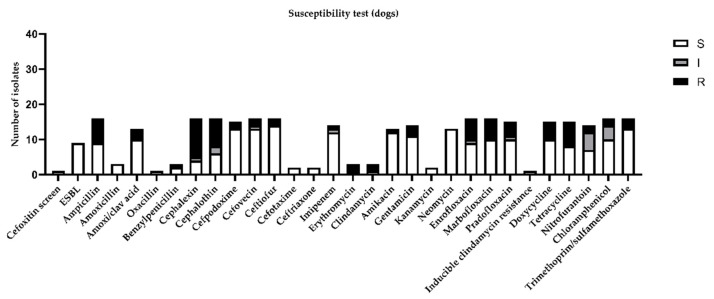
Figure represents sensitivity (S), intermediate (I), or resistance (R) to different antimicrobial drugs tested in susceptibility test, according to minimal inhibitory concentration (MIC) values considered by CLSI guidelines [11].

Considering the global distribution of the results obtained by the susceptibility test in canine urine samples, the highest percentage of resistance (more than 30%) was demonstrated for ampicillin, cephalexin, cephalothin, enrofloxacin, marbofloxacin, doxycycline, and tetracycline (Figure 2).

**Figure 3:**
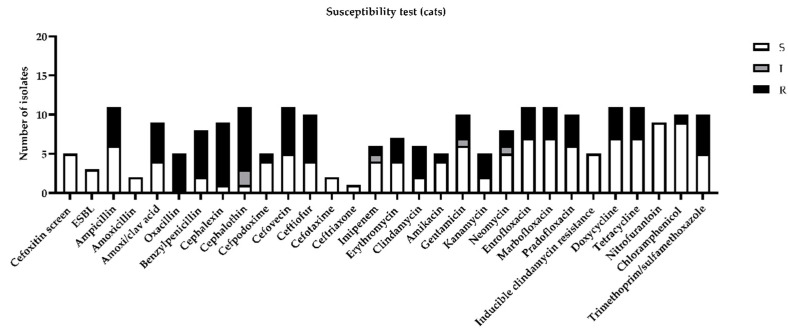
Figure represents sensitivity (S), intermediate (I), or resistance (R) to different antimicrobial drugs tested in susceptibility test, according to minimal inhibitory concentration (MIC) values considered by CLSI guidelines [11].

Among feline urine samples, the highest resistances (more than 30%) were highlighted for ampicillin, amoxicillin, clavulanic acid, benzylpenicillin, cephalexin, cephalothin, cefovecin, ceftiofur, clindamycin, enrofloxacin, marbofloxacin, pradofloxacin, doxycycline, tetracycline, and trimethoprim/sulfamethoxazole (Figure 3).

Susceptibility tests also reported on the cefoxitin screen, which is a phenotypic indication of methicillin resistance, mainly caused by the presence of mecA and mecC genes. Moreover, the susceptibility to oxacillin was also tested: All the isolated Staphylococci were positive to the cefoxitin screen and resistant to oxacillin, indicating that they were methicillin-resistant *Staphylococcus aureus* (MRSA) and *Staphylococcus pseudintermedius* (MRSP).

More specifically, only the most prevalent isolated bacteria for both species were considered: Bacteria isolated only in one sample were not considered for further comments. The percentages of sensitivity and resistance obtained by canine and feline samples are presented in Table 1 and Table 2, respectively.

### 2.3. Antibiotic Treatment

After receiving the results, a decision regarding therapy was made according to the susceptibility test, checking the lowest minimal inhibitory concentration (MIC) value. According to this, among dogs, beta-lactams (6/14), fluoroquinolones (6/14), gentamicin (1/14), and tetracycline (1/14) were prescribed. Among cats, the therapy was set with beta-lactams (6/11), fluoroquinolones (3/11), clindamycin (1/11), and nitrofurans (1/11). Antibiotic treatments are summarized in Table 3 and Table 4, for dogs and cats, respectively.

### 2.4. Patient Outcomes

The follow-up visits for the 14 dogs and the 11 cats in the present study were checked according to the scheduled control visit plan. None demonstrated side effects. All owners agreed with the control visit plan and demonstrated compliance with veterinarians and with the protocol. All of the patients were visited weekly and demonstrated an improvement in clinical conditions, with a reduction in the number of signs and symptoms of a UTI. The microbiological isolation and the susceptibility test were performed for each patient, and 13 out of 14 dogs and all the cats demonstrated etiological healing associated with a clinical improvement. Only 1 dog out of 14 was considered for recruitment since *Streptococcus canis* was still identified at a concentration of more than 1000 cfu/mL, even if the patient did not present symptoms of a UTI. The clinical and microbiological outcomes are summarized in Table 3 and Table 4 for dogs and cats, respectively.

## 3. Discussion

Establishing a rational working flow and prescribing antibiotic treatment only after receiving the antibiotic susceptibility test results seem to be a good way to treat the patient specifically and avoid recruitment. This important statement is in accordance with another recent paper, which underlined the importance of a well-designed working flow to circumscribe patient information and to choose the right antibiotic treatment consciously [3]. In the last few years, some papers were published on ASP in several countries all over the world, and guidelines were released to be adopted as national requirements and for local needs [6,14,15]. Our approach allows for the integration of international guidelines and an understanding of the level of local sensitivity involved in recording a resistance surveillance report and to prescribe a tailored therapy.

Considering the most prevalent bacteria isolated in the present study, only *E. coli*, *Proteus mirabilis*, and *Streptococcus canis* for dogs and *E. coli*, *Staphylococcus aureus*, and *Staphiloccocus pseudintermedius* for cats were considered for the discussion. In contrast with another paper [5], we considered not only MIC, but also the extended spectrum beta-lactamase (ESBL) profile cefoxitin screen and oxacillin test. All of the isolated *E. coli* were negative for ESBL and demonstrated 75% resistance against cephalothin in dogs and 66% intermediate resistance in cats. *Proteus mirabilis* in dogs demonstrated an increased rate of resistance against cephalosporins, imipenem, and neomycin. *Streptococcus canis* demonstrated 50% resistance against neomycin only. *Staphylococcus aureus* and *pseudintermedius* isolated in cat samples were all positive to the cefoxitin screen and resistant to oxacillin. According to this, they were all classified as MRSA and MRSP and were absolutely resistant to beta lactams. *Staphylococcus pseudintermedius* were totally resistant to fluoroquinolones, tetracyclines, clindamycin, and kanamycin and were partially resistant to chloramphenicol and neomycin. *Staphylococcus aureus* was partially resistant to tetracycline.

Multidrug resistance was previously defined as non-intrinsic resistance to three or more of the antimicrobial categories, but methicillin-resistant staphylococci are considered MRD, even if they are susceptible to other categories of antibiotics. According to this statement, not one of the bacteria isolated in the present study could be considered MDR [5,16].

It could, therefore, be argued that only a few cases were enrolled in the present study. This is a relative limitation, since only one hospital was considered, and the observational period lasted for only one year: Other papers reported a multicentric enrollment over a very long period (up to ten years) [10,11,17]. A previous study by an Italian referral laboratory enrolled 243 cases in five years, which equates to 48.6 patients per year [10]. We applied our protocol in a small clinical animal hospital, which is a reference center for emergencies. Moreover, our study was run during the COVID-19 pandemic, and we continued to enroll patients in February and March 2020, when Italy became the first European country to face the pandemic emergency.

In the authors’ opinion, this study boasts multiple strengths.

(i) The study was conceived considering a prospective design: A working flow was previously established with the entire medical staff in order to pursue antimicrobial stewardship. The aim was to have an objective approach that was able to limit bias and to show an example of antimicrobial stewardship that is also achievable and applicable with small groups of animals while considering a specific pathology, such as a UTI in this case. The authors agree that further enrollment of patients according to the present working flow to monitor the progression of AMR is necessary.
(ii) According to sample collection, voluntary voiding was not permitted. It is known that misinterpretation due to contaminated bacteria occurs; thus, the collection method might affect the quantity and the quality of isolates [6,17,18,19].
(iii) Samples were delivered in few hours and arrived in the laboratory in 24 h at controlled temperature conditions: This is a milestone that avoids both false positive and false negative cultures due to delayed deliveries [6,20].
(iv) Contrary to other studies [14,21], it was decided a priori not to begin an empiric treatment as routine practice but to prescribe the treatment only at the end of the working flow. This could allow for a reduction in the amount of bias linked to in-house testing, a high operator-dependent variability and, to limit risk, avoiding an attitude that is common in some cases [3]. Bacterial culture could be a good tool in decisional processes which obviously entail withholding antibiotic therapy: This condition could be acceptable if the results of culture testing are available within a short period, if no life-threatening conditions or severe clinical signs occur, and if detrimental effects on outcomes are not induced [6].
(v) The workflow was decided by medical staff with the compliance of the entire clinical staff: This approach enhances the common ownership of the ASP. This aspect was underlined by Guardabassi and Prescott [2], who also explained the importance of having an “ASP team”. We tried to establish a similar protocol, defining internal responses to infectious disease, in connection with specialists in veterinary pharmacology and with a laboratory capable of carefully and quickly processing the samples. The ASP team should be able to write specific guidelines, according to national and international regulations, dividing drugs with specific pharmacokinetic and pharmacodynamic information that are related to the different pathologies. These guidelines should be revised and updated every year and tailored to specific contexts [2,14,22].
(vi) In our study, we evaluated the clinical outcomes of all enrolled patients. This aspect, in our opinion, gives strength to our ASP, and it is in line with what is proposed by other authors [6,10] and differs from recent papers [5,11,21], which did not record any information about patient follow-up. We think that this is important as a means of completing the general picture and to correlate microbiological results with clinical data.

Since April 2019, Italy adopted a computerized method for drug prescription. This is an important tool for all veterinarians, as the integrated handbook can provide suggestions (e.g., posology or, considering food-producing animals, withdrawal period). Moreover, this system can suggest outcomes of the prescription and consumption of antimicrobial drugs, divided by species, pathology, apparatus, and type of drugs. The papers of Lehner et al. [23] and Hubbuc et al. [15] demonstrated that, in Switzerland, the introduction of the online antimicrobial stewardship program, which gives advice/recommendations, significantly decreased the prescription of antimicrobials for UTIs in dogs and cats. In the complex context of ASP, this kind of tool could be enrolled among “educational” tools. Moreover, monitoring the national attitude for prescribing drugs is a useful tool.

It was previously widely described that traditional antimicrobial treatment increased resistance phenomena in the bacteria responsible for UTIs in dogs and cats [10]. It is quite commonly reported that dogs and cats presenting signs of lower UTIs, and referred to first-line opinion veterinarians, received empirical treatment with antimicrobial drugs, even if these signs were not caused by bacterial infection [5,14]. Laboratory tests, such as microscopy, culture, and antimicrobial susceptibility tests, are usually performed in the case of relapse after a first empirical therapeutical attempt [4,24]. It is possible to find in the literature papers corroborating that it is possible to start empirical treatment with trimethoprim and sulfamethoxazole or amoxicillin in patients with UTIs due to the fact that 80% of cases still demonstrate susceptibility to these drugs [5,14]. It was also reported that 29% of dogs empirically treated for UTIs required a variation of pharmacological strategy, mainly due to re-evaluation after obtaining susceptibility test results [3]. According to this information, it is important to evaluate the application of empirical treatment carefully because a high percentage of cases is not sensitive to this therapy, or it could indue selective pressure, leading to and increasing resistance. In 2017, Rampacci and colleagues [11] considered, in a retrospectively designed study, the impact of empirical treatment in canine and feline UTIs in Italy. According to their results, the empirical treatment should not be started prior to receiving of the microbiological assay results due to the high risk to select resistant bacteria that are potentially dangerous for humans and animals. However, they did record patient outcomes and underlined the importance of developing appropriate therapeutic protocols. The data obtained by Rampacci and co-workers are partially in agreement with those recorded in our study, considering that *E. coli* was the most prevalent microorganism in dogs. That being said, we demonstrated that *Staphylococcus aureus* and *pseudintermedius* have a high prevalence in cats.

The high percentage of antimicrobial resistance for certain molecules observed in the present study could be explained by the fact that the hospital where patients were enrolled is a referral center for more complex or urgent clinical cases. Consequently, patients came from other small practices where improper drug use or empirical treatment might happen. This variable was previously reported on by other authors [11,19].

An improvement to our working-flow will be to reduce or avoid the prescription of drugs belonging to the upper classes of the Antimicrobial Advice Ad Hoc Expert Group (AMEG) classification, or those encoded in the critically important antimicrobials (CIAs) list [1,9]. In the present study, according to laboratory reports and clinical conditions, fluoroquinolones were prescribed. In the future, we aim to improve the prescription of nitrofuran, such as nitrofurantoin, which was demonstrated to have a very low percentage of resistance in the samples analyzed for the present paper, which could be effective against a wider group of the pathogens responsible for UTIs (*E. coli*, *Klebsiella*, *Enterobacter*, *Enterococci*, *Staphylococcus aureus* and *epidermidis*, *Salmonella*, *Shigella*, and *Corynebacterium*) [25,26].

## 4. Materials and Methods

### 4.1. Study Design

**Figure 4:**
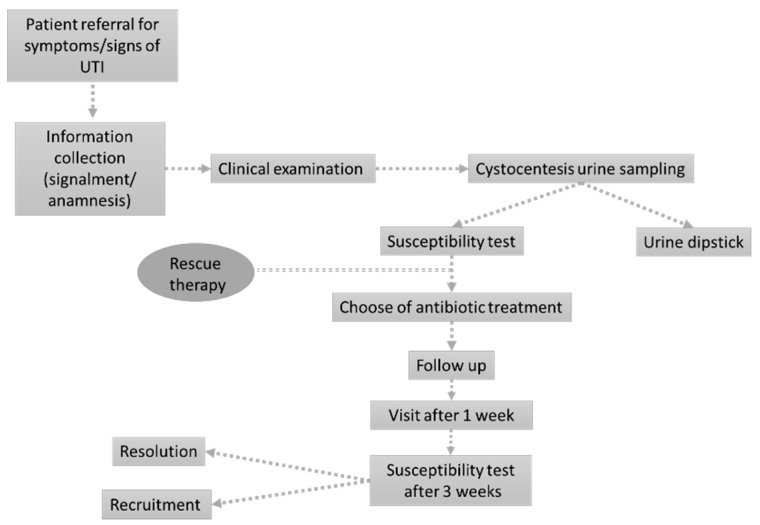
The figure represents the working flow specifically designed for the present study.

The study was designed as a prospective single-center observational study in a small animal hospital in Northwest Italy. The working flow was inspired by previously published methods [27,28] with slight modifications to enroll only patients presenting acute UTI signs and to consider both dogs and cats. The definitive working flow adopted in the present study is presented in Figure 4.

Informed consent was obtained from all owners, who gave permission to use the collected data and clinical information.

### 4.2. Data Collection

Animals were recruited from January to December 2020. On the day of consultation, patient signalment, clinical history, all clinical and diagnostic procedures, and previous pharmacological treatments were recorded.

### 4.3. Study Population

Dogs and cats of any breed, sex, and age, and presenting specific clinical signs of UTIs (stranguria, pollakiuria, hematuria) associated with indirect signs (licking, anorexia, depression) were enrolled.

### 4.4. Urine Samples and Microbiological Assays

All urine samples were collected by cystocentesis in sterile silicone-coated tubes and were sent to the laboratory to perform bacteria isolation and antibiotic-susceptibility tests, according to the Clinical & Laboratory Standards Institute (CLSI) guidelines (VET08, 2018) [24,29]. A cut-off value of more than 1000 colony forming units (CFU)/mL was considered to be clinically significant.

### 4.5. Antibiotic Treatment

Antibiotic treatment prior to receiving the antibiotic susceptibility test results was avoided, but rescue therapy using amoxicillin and clavulanic acid in the case of life-threatening conditions was permitted while waiting for the results. After receiving the results, antibiotic treatment was chosen, according to the MIC value. The prescription was written using the electronic method and respecting the posology, without changes.

### 4.6. Outcomes

A follow-up of all patients was scheduled, according to a control visit plan that encompassed a clinical check one week after the beginning of the therapy and a susceptibility test three weeks after the last day of drug administration. During the visits, general health status, monitoring of the correct drug administration, and the collection of information by the owners were recorded. These data were used to evaluate clinical and microbiological cures, and to assess the effectiveness of all antimicrobial treatments [30].

### 4.7. Data Management and Statistical Analysis

Data were organized using Excel software (Microsoft, Redmond, WA, USA), and descriptive statistics were performed using Prism 9.0 software (GraphPad, San Diego, CA, USA).

## 5. Conclusions

Several papers dealt with the problem of antimicrobial resistance in veterinary medicine using a retrospective approach. Most of them demonstrated the necessity of improving clinical practice to obtain a tailored antibiotic treatment. Considering these studies, we tried to propose a new working flow that is easy to reproduce and to explain to owners. Our experience was positive and allowed for a rational approach and a high success rate, limiting prescription and the use of empirical treatment. These findings underline the contribution of veterinary medicine on the management of antimicrobial resistance and the need for veterinarians to prescribe and use antimicrobial drugs in a careful way, without impacting animal welfare. To do so, antimicrobials should be prescribed and used only if necessary, choosing the appropriate molecule for the causative strain.

## Figures and Tables

**Table 1:** Table summarizes most representative bacteria isolated from urine samples in dogs (*n* = 14). Legend: S = sensitive, I = intermediate, R = resistant. Bacteria were ranked according to minimal inhibitory concentration (MIC) values presented in CLSI guidelines [11].

Drug	*E. coli* (8/14)			*Proteus mirabilis* (2/14)			*Streptococcus canis* (2/14)		
	S (%)	I (%)	R (%)	S (%)	I (%)	R (%)	S (%)	I (%)	R (%)
Ampicillin	50	-	50	-	-	-	-	-	-
Amoxicillin	-	-	-	-	-	-	100	-	-
Amoxicillin/ clavulanic acid	62.5	-	37.5	100	-	-	-	-	-
Benzylpenicillin	-	-	-	-	-	-	100	-	-
Cephalexin	-	-	100	-	50	50	100	-	-
Cephalothin	12.5	12.5	75	50	50	-	100	-	-
Cefpodoxime	100	-	-	100	-	-	100	-	-
Cefovecin	100	-	-	50	50	-	100	-	-
Ceftiofur	100	-	-	-	-	100	-	-	100
Cefotaxime	-	-	-	-	-	-	100	-	-
Ceftriaxone	-	-	-	-	-	-	100	-	-
Imipenem	100	-	-	-	-	-	-	-	-
Eritromycin	-	-	-	-	-	100	100	-	-
Amikacin	100	-	-	100	-	-	-	-	-
Gentamycin	75	-	25	100	-	-	-	-	-
Neomicyn	75	-	25	50	50	-	50	50	-
Enrofloxacin	75	-	25	50	-	50	50	50	-
Marbofloxacin	75	-	25	50	-	50	100	-	-
Pradofloxacin	75	-	25	-	-	100	50	-	50
Doxyciclyne	75	-	25	-	-	100	100	-	-
Tetracycline	75	-	25	-	-	100	-	-	100
Nitrofurantoin	50	50	-	-	-	100	-	-	-
Chloramphenicol	25	50	25	100	-	-	-	-	-
Trimethoprim/ Sulfamethoxazole	75	-	25	100	-	-	100	-	-
ESBL	neg			Not evaluated			Not evaluated		

**Table 2:** Table summarizes most representative bacteria isolated from urine samples in cats (*n* = 11). Legend: S = sensitive, I = intermediate, R = resistant. Bacteria were ranked according to minimal inhibitory concentration (MIC) values presented in CLSI guidelines [11].

Drug	*E. coli* (3/11)			*Staphylococcus pseudintermedius* (3/11)			*Staphylococcus aureus* (2/11)		
	S (%)	I (%)	R (%)	S (%)	I (%)	R (%)	S (%)	I (%)	R (%)
Ampicillin	100	-	-	-	-	100	-	-	100
Amoxicillin/ clavulanic acid	100	-	-	-	-	100	-	-	100
Oxacillin	-	-	100	-	-	100	-	-	100
Benzylpenicillin	-	-	100	-	-	100	-	-	100
Cephalexin	-	-	100	-	-	100	-	-	100
Cephalothin	-	66	34	-	-	100	-	-	100
Cefpodoxime	100	-	-	-	-	-	-	-	-
Cefovecin	100	-	-	-	-	100	-	-	100
Ceftiofur	100	-	-	-	-	100	-	-	100
Imipenem	100	-	-	-	-	-	-	-	-
Eritromycin	-	-	-	-	-	100	100	-	-
Clindamycin	-	-	-	-	-	100	50	-	50
Amikacin	100	-	-	-	-	-	-	-	-
Gentamycin	100	-	-	-	34	66	100	-	-
Kanamicin	-	-	-	-	-	100	100	-	-
Neomicyn	100	-	-	-	34	66	100	-	-
Enrofloxacin	100	-	-	-	-	100	50	-	50
Marbofloxacin	100	-	-	-	-	100	50	-	50
Pradofloxacin	100	-	-	-	-	100	50	-	50
Inducible clyndamicin resistance	-	-	-	neg			neg		
Doxyciclyne	100	-	-	-	-	100	50	-	50
Tetracycline	100	-	-	-	-	100	50	-	50
Nitrofurantoin	100	-	-	100	-	-	100	-	-
Chloramphenicol	100	-	-	66	-	34	100	-	-
Trimethoprim/ Sulfamethoxazole	100	-	-	-	-	100	100	-	-
ESBL	neg			Not evaluated			Not evaluated		
Cefoxitin screen	Not evaluated			pos			pos		

**Table 3:** Table summarizes drugs used to treat dogs according to results of susceptibility test, frequency of the different treatments considering total number of enrolled dogs (14), bacteria isolated in urine samples, clinical and the microbiological outcomes considering number of dogs that received that specific therapy.

Therapy	Frequency (*n* = 14)	Bacteria	Outcomes	
			Clinical Cure	Microbiological Cure
Fluoroquinolones	6/14	*Staphylococcus pseudointermedius*, *Streptococcus canis*, *E. coli*	Yes (6/6)	Yes 5/6 No 1/6 (recruitment *Streptococcus canis*)
Beta lactams	6/14	*Enterococcus faecalis*, *E. coli*	Yes (6/6)	Yes (6/6)
Doxycycline	1/14	*Klebsiella Pneumoniae*	Yes	Yes (1/1)
Gentamicin	1/14	*Proteus mirabilis*	Yes	Yes (1/1)

**Table 4:** Table summarizes drugs used to treat cats according to results of susceptibility test, frequency of different treatments considering total number of enrolled cats (11), bacteria isolated in urine samples, clinical and microbiological outcomes considering number of cats that received that specific therapy.

Therapy	Frequency (*n* = 11)	Bacteria	Outcomes	
			Clinical Cure	Microbiological Cure
Beta lactams	6/11	*Corynebacterium aurimucosum*, *E. coli*, *Staphylococcus aureus*	Yes (6/6)	Yes (6/6)
Fluoroquinolones	3/11	*Staphylococcus pseudointermedius*, *Enterococcus faecalis*	Yes (3/3)	Yes (3/3)
Nitrofuratoin	1/11	*Staphylococcus pseudointermedius*,	Yes (1/1)	Yes (1/1)
Clindamicycin	1/11	*Staphylococcus aureus*	Yes (1/1)	Yes (1/1)
